# Older Adults’ and Clinicians’ Perspectives on a Smart Health Platform for the Aging Population: Design and Evaluation Study

**DOI:** 10.2196/29623

**Published:** 2022-02-28

**Authors:** Alessia Cristiano, Stela Musteata, Sara De Silvestri, Valerio Bellandi, Paolo Ceravolo, Matteo Cesari, Domenico Azzolino, Alberto Sanna, Diana Trojaniello

**Affiliations:** 1 Center for Advanced Technology in Health and Wellbeing Istituto di Ricovero e Cura a Carattere Scientifico Ospedale San Raffaele Milan Italy; 2 Department of Computer Science Università Degli Studi di Milano Milan Italy; 3 Geriatric Unit Istituto di Ricovero e Cura a Carattere Scientifico Istituti Clinici Scientifici Maugeri Università Degli Studi di Milano Milan Italy

**Keywords:** smart health, remote monitoring, requirement elicitation, older population, age-related chronic conditions, healthy aging, Internet of Things, mobile phone

## Abstract

**Background:**

Over recent years, interest in the development of smart health technologies aimed at supporting independent living for older populations has increased. The integration of innovative technologies, such as the Internet of Things, wearable technologies, artificial intelligence, and ambient-assisted living applications, represents a valuable solution for this scope. Designing such an integrated system requires addressing several aspects (eg, equipment selection, data management, analytics, costs, and users’ needs) and involving different areas of expertise (eg, medical science, service design, biomedical and computer engineering).

**Objective:**

The objective of this study is 2-fold; we aimed to design the functionalities of a smart health platform addressing 5 chronic conditions prevalent in the older population (ie, hearing loss, cardiovascular diseases, cognitive impairments, mental health problems, and balance disorders) by considering both older adults’ and clinicians’ perspectives and to evaluate the identified smart health platform functionalities with a small group of older adults.

**Methods:**

Overall, 24 older adults (aged >65 years) and 118 clinicians were interviewed through focus group activities and web-based questionnaires to elicit the smart health platform requirements. Considering the elicited requirements, the main functionalities of smart health platform were designed. Then, a focus group involving 6 older adults was conducted to evaluate the proposed solution in terms of usefulness, credibility, desirability, and learnability.

**Results:**

Eight main functionalities were identified and assessed—cognitive training and hearing training (usefulness: 6/6, 100%; credibility: 6/6, 100%; desirability: 6/6, 100%; learnability: 6/6, 100%), monitoring of physiological parameters (usefulness: 6/6, 100%; credibility*:* 6/6, 100%; desirability: 6/6, 100%; learnability: 5/6, 83%), physical training (usefulness: 6/6, 100%; credibility: 6/6, 100%; desirability: 5/6, 83%; learnability: 2/6, 33%), psychoeducational intervention (usefulness: 6/6, 100%; credibility: 6/6, 100%; desirability: 4/6, 67%; learnability: 2/6, 33%), mood monitoring (usefulness: 4/6, 67%; credibility: 4/6, 67%; desirability: 3/6, 50%; learnability: 5/6, 50%), diet plan (usefulness: 5/6, 83%; credibility: 4/6, 67%; desirability*:* 1/6, 17%; learnability: 2/6, 33%), and environment monitoring and adjustment (usefulness: 1/6, 17%; credibility: 1/6, 17%; desirability: 0/6, 0%; learnability: 0/6, 0%). Most of them were highly appreciated by older participants, with the only exception being environment monitoring and adjustment. The results showed that the proposed functionalities met the needs and expectations of users (eg, improved self-management of patients’ disease and enhanced patient safety). However, some aspects need to be addressed (eg, technical and privacy issues).

**Conclusions:**

The presented smart health platform functionalities seem to be able to meet older adults’ needs and desires to enhance their self-awareness and self-management of their medical condition, encourage healthy and independent living, and provide evidence-based support for clinicians’ decision-making. Further research with a larger and more heterogeneous pool of stakeholders in terms of demographics and clinical conditions is needed to assess system acceptability and overall user experience in free-living conditions.

## Introduction

### Background

Currently, 22% of the total population in Europe is aged >65 years, and this number is estimated to increase to 51% by 2070 [1]. More than 50% of the existing older adults have ≥3 chronic disorders (eg, hypertension, heart disease, and diabetes) that negatively affect their quality of life (QoL) and independent living [2]. The multiple comorbidities of chronic conditions with co-occurring age-related cognitive and behavioral changes make older adults frail, with a consequent increased risk of geriatric syndromes, hospitalization, and disability. Hence, the aging population is expected to be a great challenge for health care systems and represents a perfect target for developing new smart health platforms [3].

Smart health is a concept that refers to the multidimensional change that medical care is facing as a result of the integration of mobile devices (eg, smartphones), wearables (eg, fitness bands), and smart medical devices (eg, smart blood pressure monitors). These instruments enable the collection of massive amounts of health-related data that, when analyzed with artificial intelligence models, can provide insights for a personalized intervention. Smart health systems have become increasingly feasible in recent years because of the remarkable technological advancements in processing power, network infrastructures, and big data analytics, leading to a high level of information processing [4]. Big data may offer many advantages in the health care sector [4-6]: they are decisive in the prevention and early detection of diseases, risk monitoring, definition of tailored interventions based on a patient-centered over a disease-centered approach, objective reporting and evidence-based medicine, reduction of social and medical costs, and public health surveillance.

Over the past decade, the needs of the aging society have been investigated, and several smart health solutions have been proposed. The ultimate goal of smart health systems devoted to the older population is to encourage healthy lifestyles, increase autonomy, facilitate social inclusion, guarantee continuity of medical therapy even at home, and provide remote monitoring and teleconsulting. Technologies capable of monitoring an individual’s activities and behaviors [7-9] have been developed to promote healthy habits (eg, active living and healthy nutrition). For better management of age-related diseases, devices supporting a proper intake of medication [10,11] have been suggested, and mobile apps providing remote monitoring [5], cognitive training [12], and psychological support [13-15] have been proposed. In addition, systems enabling the monitoring of home environmental conditions [16,17] or the detection of falls [9,18] have been designed to increase the safety of older adults.

The use of smart health solutions by the older population has been widely explored in the current literature [19-22]. In that regard, the attractiveness, ease of use (eg, understandable and simple language and easy access to information), and perceived added value of the technology (eg, relevant and valuable functionalities) are considered facilitators of the adoption of new technology for older adults [21,23]. Other enablers lie in an individual’s adequate education to the use of technology because of prior experience with digital devices and mobile apps and in the curiosity toward the new technology [23]. The presence of support for older adults in learning to use the technology positively predisposes them to the use of new technological solutions [23]. In contrast, the literature suggests that the main barriers to the prolonged use of technology by older adults are issues in the usability of the system and perceived irrelevance of an application or a device with a resulting sense of the uselessness of the entire technology [23]. Moreover, the physical and functional age (ie, a combination of physiological, psychological, and social age determined by measures of functional capability indexed by age-normed standards), the absence of instructions or guidance, computer anxiety, and lack of confidence can lead to premature abandonment of those solutions by older populations [21,24,25]. Furthermore, relative to their use, older adults expressed privacy concerns, disapproval of possible excessive control from the caregivers, and lack or reduction of social interaction [20,21].

An important step in the design process of technology for community-dwelling older adults is to collect and address the needs of all involved stakeholders (eg, older patients, caregivers, and clinicians) [19,20,26]. A recent study [27] on a telepsychiatry service suggested that clinicians’ concerns must be considered and addressed in the design and development of a service targeted for older adults. Another work [28] highlighted that poor involvement of the health care team in the development of an assistance and intervention service leads to reduced treatment adherence for patients [28]. Hence, all end users have to be involved in the design and implementation phases of a smart health platform for the older population.

However, research exploring attitudes, perceptions, expectations, and concerns about smart health technologies of both older adults and clinicians is limited, and users’ well-being is often treated as a secondary outcome by assistive technology designers [29]. Moreover, the focus of most studies has been on exploring a single device (eg, tablet) [21] or function (eg, telemonitoring of daily activities) [20,26,27] and not a platform or a system including different devices and services.

### Objective

This study, conducted within the Horizon 2020 European project *SMART BEAR*, addresses the aforementioned limitations. The idea underpinning this project is the implementation of an affordable, accountable, and privacy-preserving innovative platform (ie, SMART BEAR platform), integrating off-the-shelf smart and medical devices. The focus of developing such a platform is to support the healthy and independent living of aging people with five prevalent health-related conditions: hearing loss, cardiovascular diseases, cognitive impairments, mental health issues, and balance disorders. For every medical condition, the platform intends to provide the end users with remote monitoring and intervention based on several functionalities that may improve their QoL and facilitate disease management. More specifically, the platform aims to fulfill five objectives: (1) promote patients’ self-awareness of health status, (2) promote patients’ self-management of their own health conditions, (3) encourage patients’ active living, (4) enable patient’s independent living, and (5) provide evidence-based support for clinicians’ decision-making.

Within this context, this study aims to collect key requirements for the SMART BEAR platform design by involving both older adults and clinicians. Specifically, the objective of the study is 2-fold; we aimed to understand stakeholders’ beliefs, attitudes, needs, expectations, and concerns about the SMART BEAR platform (*objective 1*) and to evaluate the proposed solution with a small group of older adults (*objective 2*). The paper is structured as follows: the methodology, methods, data collection, sample population, and data analysis are described in the *Methods* section; results obtained are reported in the *Results* section; and discussion on the insights gained is narrated in the *Discussion* section. The paper ends with the conclusions, limits, and future work being presented.

## Methods

### Overview

A 2-phase experimental procedure was designed to address the objectives of this study. The first phase (*requirements collection*) was devoted to gaining a comprehensive understanding of the behaviors and perceptions of both clinicians and older adults, as well as exploring environmental factors that influence their adoption of the technology (*objective 1)*. Once this process was completed and the collected data were analyzed, the main functionalities to make the SMART BEAR platform effective and adoptable by the end users could be identified. The second phase (*evaluation*) was intended to provide an overall assessment of the designed platform and its functionalities (*objective 2*).

### Methodology

The methods used to gather clinicians’ and older adults’ data can be distinguished into two categories: *focus group* and *web-based questionnaire*.

A *focus group* activity can be defined as a discussion within a small group of people (eg, 4-10 participants) about a specific topic led by a well-trained facilitator (eg, a psychologist or a researcher able to stimulate an active engagement of participants in the debate). Although it is a time-consuming activity, the focus group is well-appreciated in medical research as it represents a valid method for collecting qualitative and quantitative information. Conversely, *web-based questionnaires* allow gathering information from a large sample in a short period; it is easy to fill in remotely using a computer or a smartphone, and its answers are simple to analyze as a more structured survey.

To establish the content and structure of the methods used in the study, a draft of questions was first created according to preliminary informal interviews conducted with experts (eg, neuropsychologists, geriatricians, and engineers). Then, a brainstorming session was conducted to decide which questions to include or exclude (eg, “Is this question really needed?”). The brainstorming was helpful in avoiding the temptation to include questions without critical evaluation of their contribution toward the achievement of the study objectives. Finally, special attention was given to the wording, length, order, and format of questions (eg, several factors such as the age of the target respondents were considered, and the font size of the questionnaires was adapted accordingly). The questions were organized and worded to encourage respondents to provide accurate, unbiased, and complete information.

### Requirements Collection Phase

The *requirements collection* phase included three subsequent activities: first, a focus group activity with clinicians (*focus group for clinicians*) was conducted to collect qualitative exploratory information for a better understanding of how the SMART BEAR platform can benefit older adults and their physicians. Clinicians with various medical specialties (eg, geriatricians, cardiologists, psychiatrists, neurologists, and psychologists) were encouraged to participate in the activity. Indeed, their experience with older patients and their caregivers may offer valuable perspectives on the problems faced in clinical practice and how the technology may facilitate the management of prevalent age-related conditions. Moreover, they were invited to debate about the *intrinsic capacity* (IC) model [30] introduced by the World Health Organization, according to which the individual’s functional abilities need to be considered to ensure a comprehensive characterization of older patients. Second, a structured questionnaire was issued via the web to a large sample of clinicians (*web-based questionnaire for clinicians*) to learn about their beliefs, attitudes, and expectations on the SMART BEAR platform. In the third phase, a web-based questionnaire was set up and disseminated among older adults (*web-based questionnaire for older adults*) to collect their feedback and impressions about the SMART BEAR platform.

### Evaluation Phase

The *evaluation* phase included a focus group activity with older adults using a storytelling approach (*focus group for older adults*). The participants, as potential users of the SMART BEAR platform, were invited to answer structured questions while observing archetypal users (ie, users with similar age and clinical conditions) experiencing the proposed technological solution and its functionalities.

### Experimental Procedure

During the *requirements collection* phase, once the participants’ demographic data were gathered, 5 areas were investigated overall through the *focus group for clinicians*, *web-based questionnaire for clinicians,* and *web-based questionnaire for older adults* (Table 1). In detail, in the *focus group for clinicians*, the facilitator (ie, a neuropsychologist) explored four areas of interest for clinicians (ie, impact of disease in everyday life, remote monitoring, use of technology in medical practice, and about SMART BEAR) by administering a set of 33 open-ended questions to the participants (Multimedia Appendix 1). Instead, the *web-based questionnaire for clinicians* comprised 13 closed, multiple-choice questions to guarantee clarity, brevity, and usability of the questionnaire, given its web-based nature. The questions were selected from among those used in the *focus group for clinicians*. They covered 3 of the 4 areas of clinicians’ interest (ie, impact of disease in everyday life, being a very broad and complex topic, was excluded to avoid an excessive workload for the respondents).

Furthermore, free-form comment boxes were added to gather further participants’ insights. The *web-based questionnaire for clinicians* (Multimedia Appendix 1) was published on the Limesurvey platform, and its link was spread through the internal mailing lists of Istituto di Ricovero e Cura a Carattere Scientifico (IRCCS) Policlinico Ca’ Granda (Milan, Italy), Ospedale Maggiore (Crema, Italy), and IRCCS Ospedale San Raffaele (Milan, Italy). Similarly, for the *web-based questionnaire for older adults*, the multiple-choice, close-ended questions structure was preferred, and 35 questions were selected to cover the areas targeted for older adults (ie, impact of disease in everyday life, remote monitoring, older adults’ relationship with technology, and about SMART BEAR). The questionnaire for older adults (Multimedia Appendix 1) was published on the Limesurvey platform, and the link was shared among the contacts of clinicians, colleagues, and older participants of previous research projects.

**Table 1:** Investigated areas and used methods for each phase.

Phase			Clinicians		Older adults
**Requirements collection phase (areas)**					
	Impact of disease in everyday life	Focus group		Web-based questionnaire	
	Remote monitoring	Focus group and web-based questionnaire		Web-based questionnaire	
	Older adults’ relationship with the technology	N/A^a^		Web-based questionnaire	
	Use of technology in medical practice	Focus group and web-based questionnaire		N/A	
	About SMART BEAR	Focus group and web-based questionnaire		Web-based questionnaire	
**Evaluation phase (interventions)**					
	Physical training	N/A		Focus group: S_1_^b^	
	Diet plan	N/A		Focus group: S_1_	
	Monitoring of physiological parameters	N/A		Focus group: S_1_	
	Psychoeducational intervention	N/A		Focus group: S_2_^c^	
	Monitoring of the mood	N/A		Focus group: S_2_	
	Cognitive training	N/A		Focus group: S_2_	
	Hearing training	N/A		Focus group: S_2_	
	Environment monitoring and adjustment	N/A		Focus group: S_2_	
**Evaluation phase (transversal functions)**					
	Data visualization	N/A		Focus group: S_1_ and S_2_	
	Gamification	N/A		Focus group: S_1_ and S_2_	
	Regular report	N/A		Focus group: S_1_ and S_2_	
	Regular report to clinician	N/A		Focus group: S_1_ and S_2_	
	Suggestion	N/A		Focus group: S_1_ and S_2_	
	Reminder	N/A		Focus group: S_1_ and S_2_	
	Data access to caregiver	N/A		Focus group: S_1_ and S_2_	
	Teleconsulting	N/A		Focus group: S_1_ and S_2_	

^a^N/A: not applicable.

^b^S_1_: Carlo’s story.

^c^S_2_: Lidia’s story.

According to the data collected in the *focus group for clinicians*, *web-based questionnaire for clinicians,* and *web-based questionnaire for older adults* and their analysis, 8 interventions and 8 transversal functions of the SMART BEAR platform were proposed and assessed in the *evaluation* phase through the *focus group for older adults’* activity (Table 1)*.* It comprised a discussion based on a narration where the contents of the research questions are merged with the story of personas (ie, archetypal users). This method was selected as it encourages the identification of the participants with the protagonist, which facilitates the comprehension of the proposed technology use. Moreover, it enables participants to bring new ideas and personal insights into the discussion. In more detail, 2 stories (Carlo’s story and Lidia’s story; Multimedia Appendix 1), describing 2 personas (ie, Carlo and Lidia) interacting with the platform and making use of specific interventions and transversal functions, were presented and discussed. More specifically, in each story, different interventions were illustrated according to the protagonist’s problems (eg, physical training is offered in Carlo’s story as Carlo conducts a sedentary lifestyle). The presented interventions were evaluated in terms of usefulness (ie, *Do you find it useful to meet your needs*?), credibility (ie, *Do you think or feel it credible*?), desirability (ie, *Would you find it desirable*?), and learnability (ie, *Would you be able to learn to use it*?). Transversal functions were presented in both stories because of their versatility and evaluated in terms of usefulness and desirability.

### Sample Population

**Figure 1:**
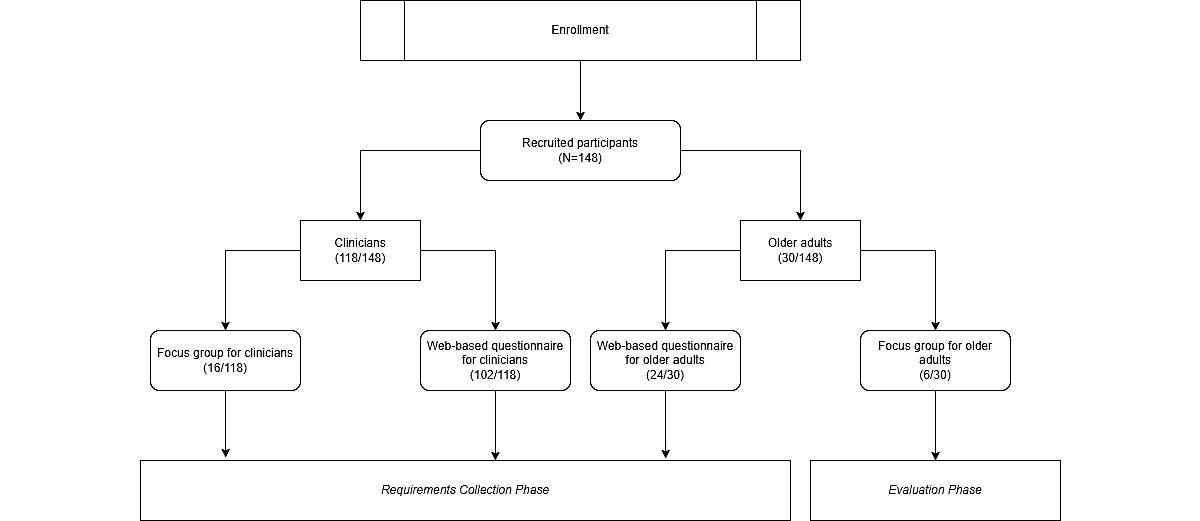
Sample population diagram.

An overall sample of 148 participants (Figure 1), comprising both clinicians (118/148, 79.7%) and older adults (30/148, 20.3%), took part in the study. The research was designed in accordance with the European Union Guidelines for Clinical Practice and the current revision of the Declaration of Helsinki. The study was approved by the Ethics Committee of the University of Milan (nr. 50.20 on May 14, 2020). All participants provided informed written consent before enrollment in the study.

As shown in Figure 1, clinicians (16/118, 13.6%) with expertise in all medical domains from two hospitals, IRCCS Policlinico Ca’ Granda and Ospedale Maggiore, were involved in the *focus group for clinicians.* A focus group was planned in each hospital. More specifically, 56% (9/16) of participants took part in the first focus group at Policlinico Ca’ Granda (*focus group 1 for clinicians*), whereas the second focus group was performed with the involvement of 44% (7/16) of medical experts at Ospedale Maggiore in Crema (*focus group 2 for clinicians*). Each focus group lasted approximately 90 minutes and was conducted in a specifically furnished room at the hospital’s premises. The focus groups were led by a neuropsychologist, whereas 2 biomedical engineers took notes.

Physicians (102/118, 86.4%) with expertise in ≥1 of the medical condition of interest were enrolled from IRCCS Policlinico Ca’ Granda, Ospedale Maggiore, and IRCCS San Raffaele Hospital for the following step of the study (ie, filling the *web-based questionnaire for clinicians*).

Participants aged ≥65 years with any of the target age-related conditions were recruited for the last 2 activities through word-of-mouth communication. In particular, of the 30 older adults, 24 (80%) participated in the *web-based questionnaire for older adults’* activity in the *requirements collection* phase, and 6 (20%) took part in the last *focus group for older adults’* activity in the *evaluation* phase.

The *focus group for older adults* was conducted by a neuropsychologist and 2 bioengineers in a medical office in Chiesa in Valmalenco (Sondrio, Italy) and lasted approximately 90 minutes. More specifically, the neuropsychologist conducted the discussion of the topics, and the 2 bioengineers took notes of the discussions and occasionally intervened to obtain a better explanation of concepts that emerged from the discussion. Users’ comments and feedback were collected in dedicated sheet forms.

### Data Analysis

The collected data were digitalized (*focus group for clinicians* and *focus group for older adults*) or exported (*web-based questionnaire for clinicians* and *web-based questionnaire for older adults*) in Microsoft Excel (Microsoft Corporation).

A separate content analysis was conducted on the qualitative data gathered in the *requirements collection* phase (*focus group for clinicians*, *web-based questionnaire for clinicians,* and *web-based questionnaire for older adults*). Every answer was assigned to a group of common opinions or preferences to allow the conversion of qualitative information into quantitative data. Then, the quantitative data collected in the *focus group for clinicians* and a *web-based questionnaire for clinicians* referring to the same questions were combined to gain the clinicians’ overall outcomes. Hence, a frequency analysis was conducted, and the results, in terms of the number of occurrences and related percentage values, were reported separately for each area investigated and involved stakeholders (clinicians or older adults).

At the end of the *requirements collection* phase analysis, researchers with heterogeneous expertise (eg, biomedical engineers, neuropsychologists, geriatric specialists, and computer scientists) formed a working group to design the functions and interventions of the smart health platform. For this purpose, the design considered the project’s objectives; namely, to address the five areas of the IC model (ie, locomotion, cognition, vitality, sensory, and psychology). The results obtained in the *requirements collection* phase allowed for the assessment of the impact of such technology on the life of the target population in terms of needs satisfaction and technology acceptance. Moreover, implementation factors were considered, resulting in the endorsement of consumer technology. The working group debated on the possible solutions to reach these objectives and finalized the design by identifying 8 interventions and 8 transversal functions, which constitute the subject for the *evaluation phase*.

Here, a content analysis was conducted for qualitative data, and a frequency analysis was conducted for quantitative data. The results obtained in that phase were reported only in terms of percentage as a small group of older adults participated in the *focus group for older adults*.

## Results

### Requirements Collection Phase

#### Focus Group for Clinicians Sample Population

A group of 16 clinicians (8/16, 50% women) between the ages of 27 and 64 years (mean 42, SD 13 years) was recruited for the *focus group for clinicians* (ie, 7/16, 44% physicians; 3/16, 19% geriatricians; 3/16, 19% cardiologists; 1/16, 6% surgeons; and 2/16, 12% medical scientists). They all agreed that high blood pressure, ischemic disease, cardiac arrhythmias, imbalance, hearing loss, falling, dementia, depression, anxiety, and stress are the principal clinical problems that challenge older adults’ everyday lives. The most treated medical conditions were high blood pressure (12/16, 75%), ischemic heart disease (10/16, 63%), arrhythmias (9/16, 56%), dementia (9/16, 56%), depression (7/16, 44%), falls (6/16, 38%), imbalance (6/16, 38%), anxiety (6/16, 38%), stress (3/16, 19%), and hearing loss (2/16, 13%).

#### Web-Based Questionnaire for Clinicians Sample Population

A sample of 102 participants completed the *web-based questionnaire for clinicians*. Approximately 98% (100/102) of the respondents expressed their area of expertise (ie, 59/102, 57.8% had expertise in geriatrics; 7/102, 6.8% had expertise in surgery; 6/102, 5.8% had expertise in general medicine; 4/102, 3.9% had expertise in neurology and physiotherapy; 2/102, 1.9% had expertise in cardiology, emergency medicine, and internal medicine; and 1/102, 0.9% had expertise in ear, nose, and throat, psychiatry, gastroenterology, pathological anatomy, urology, rheumatology, nephrology, radiology, psychology, odontology, endocrinology, oncology, and nutrition). The most frequently treated medical conditions by the surveyed clinicians were dementia (68/102, 66.6%), hypertension (64/102, 62.7%), falls (58/102, 56.8%), arrhythmias (55/102, 53.9%), ischemic heart disease (50/102, 49%), anxiety and depression (46/102, 45%), imbalance (39/102, 38.2%), stress (21/102, 20.5%), and hearing loss (9/102, 8.8%). Regarding the frequency of visits, 25.4% (26/102) of the sample declared that they visited their older patients more than once per month, 31.3% (32/102) visited every 1 to 3 months, 34.3% (35/102) visited every 6 months, and 9.8% (10/102) visited once per year.

#### Web-Based Questionnaire for Older Adults Sample Population

A total of 24 participants (16/24, 67% women) aged >65 years completed the *web-based questionnaire for older adults.* The sample was distributed as follows: 67% (16/24) in the 65 to 70 age range group, 17% (4/24) in the 71 to 75 age range group, 8% (2/24) in the 76 to 80 age range group, and 8% (2/24) in the >81 years group. Most respondents (16/24, 67%) declared that they lived with someone (all of them claimed to share their home with their spouse and 5/24, 21% with their progeny as well). The medical conditions prevalent among participants were hypertension (9/24, 38%), cardiovascular disease (6/24, 25%), anxiety (4/24, 17%), hearing difficulties (3/24, 13%), arrhythmias (4/24, 17%), balance disorders (2/24, 8%), and depression (1/24, 4%). Instead, 21% (5/24) of participants claimed to have experienced none of the abovementioned disorders.

A description of stakeholders involved in the first phase of the experimental procedure (*focus group for clinicians*, *web-based questionnaire for clinicians*, and *web-based questionnaire for older adults*) clustered according to the medical condition treated or experienced is summarized in Table 2.

**Table 2:** Requirements collection phase medical conditions treated or experienced (N=142).

Medical conditions	Clinicians (n=118), n (%)	Older adults (n=24), n (%)
Hearing loss (ie, tinnitus and unreceptiveness)	11 (9.3)	3 (12.5)
Cardiovascular diseases (ie, arrhythmias, ischemic heart disease, and hypertension)	82 (69.5)	16 (66.7)
Cognitive impairments (ie, dementia)	77 (65.3)	0 (0)
Mental health problems (ie, anxiety, depression, and stress)	68 (57.6)	4 (16.7)
Balance disorders (ie, imbalance and falls)	68 (57.6)	2 (8.3)
None	0 (0)	5 (20.8)

#### Area 1: Impact of Disease on Everyday Life

Table 3 summarizes the clinician (*focus group for clinicians*) and older adult (*web-based questionnaire for older adults*) inputs related to the *impact of disease in everyday life*.

All clinicians (16/16, 100%) agreed on the impact of age-related disorders on older adults’ daily living activities, and approximately all of them (13/16, 81%) considered that a personalized intervention was required. Approximately all participants (15/16, 94%) suggested that the family members of the older person were most affected by the onset of the disease. Specifically, according to clinicians, 88% (14/16) of caregivers report having mental health problems (eg, burnout, depression, and sleeping difficulties). Other issues encountered by caregivers in handling the patient, as observed by clinicians, are the reconciliation of their own commitments and time with the needs of the patient (eg, daily assistance, scheduling medical appointments, and bringing them to the appointments), the experience or know-how to manage the disease or the adverse clinical situations, therapy management, and supervision. The whole group (16/16, 100%) suggested that a significant barrier for older adults in their disease management is therapy compliance. Thus, the clinicians claimed that these issues have a relevant impact on caregivers’ mental health (ie, caregivers often report stress and anxiety).

Older adults reported effects on cardiorespiratory functions (6/24, 25%), sleep (6/24, 25%), and diet (3/24, 13%) for the vital area, whereas impact on sight (7/24, 29%), hearing (4/24, 17%), and smell (4/24, 17%) were observed for the sensorial area. Impacts on anxiety (7/24, 29%), depression (1/24, 4%), irritability (5/24, 21%), energy (5/24, 21%), and sociality (2/24, 8%) were found within the psychological area, whereas effects on balance (2/24, 8%), locomotion (3/24, 13%), and muscle strength (6/24, 25%) were noticed in the motor area. With regard to the cognition area, participants reported memory (10/24, 42%), attention (5/24, 21%), and language (1/24, 4%) difficulties. Most of the older adults (17/24, 71%) declared that no help was needed to manage their medical condition. However, the remaining 29% (7/24) were used to asking for help (3/24, 12% sometimes and 4/24, 17% rarely) in daily living activities (eg, personal hygiene and visiting the physician). Moreover, participants declared that they visited the clinician more than once per month (7/24, 29%), every 1 to 3 months (7/24, 29%), every 6 months (8/24, 33%), and less than once per 5 years (2/24, 8%). They joined their clinician on phone calls (18/24, 75%), written messages (10/24, 42%), and webpages (1/24, 4%).

**Table 3:** Results related to the impact of disease in everyday life (N=40).

Intrinsic capacity model areas	Clinicians (n=16), n (%)	Older adults (n=24), n (%)
Vital area (eg, cardiorespiratory functions, appetite, and autonomy)	15 (94)	13 (54)
Sensorial area (ie, vision, hearing, and smell)	6 (38)	11 (46)
Psychological area (eg, anxiety, depression, euphoria, and irritability)	13 (81)	17 (71)
Motor area (eg, balance, locomotion, coordination, and strength)	9 (56)	14 (58)
Cognitive area (ie, memory, attention, and language)	10 (63)	10 (42)

#### Area 2: Remote Monitoring

On the basis of the discussion stimulated among clinicians during the focus group, it was found that the most adopted intervention for older adults with the targeted clinical conditions seemed to be outpatient monitoring (96/118, 81.3% of clinicians), and only in some cases (30/118, 25.4% of clinicians), hospitalization was considered necessary.

Furthermore, 2.5% (3/118) of clinicians reported at the *focus group for clinicians* to have experience with remote monitoring of older patients, and the most monitored parameters were blood pressure and therapy compliance. The most commonly used means for remote communication were phone calls (74/118, 62.7%), emails (52/118, 44%), and WhatsApp communication (47/118, 39.8%). However, one of the clinicians reported feeling stressed from remote monitoring.

Regarding older adults, only 58% (14/24) of the interviewees used remote monitoring devices; in particular, 54% (13/24) of the participants claimed to use a smart blood pressure monitor, whereas 4% (1/24) stated that they used an electrocardiogram monitor. Table 4 shows the primary outcomes related to *remote monitoring* from clinicians (*focus group for clinicians and web-based questionnaire for clinicians*) and older adults (*web-based questionnaire for older adults*).

**Table 4:** Results related to remote monitoring (N=142).

Parameters considered useful			Clinicians (n=118), n (%)		Older adults (n=24), n (%)
Blood pressure			85 (72)		12 (50)
House temperature			13 (11)		1 (4.2)
Air pollution			11 (9.3)		3 (12.5)
ECG^a^			37 (31.4)		0 (0)
Fall detection			74 (62.7)		2 (8.3)
Heart rate			65 (55.1)		5 (20.8)
Glycemia			47 (39.8)		0 (0)
Social interaction frequency			42 (35.6)		2 (8.3)
Diet habits			68 (57.6)		8 (33.3)
Levels of noise exposure			13 (11)		1 (4.2)
None			0 (0)		4 (16.7)
**Other**					
	Physical activity	8 (6.8)		10 (41.7)	
	Cognitive functions	68 (57.6)		—^b^	
	Weight	1 (0.8)		—	
	Medication adherence	1 (0.8)		—	
	Behavioral changes and mood	50 (42.4)		3 (12.5)	
	Sleep quality	3 (2.5)		8 (33.3)	
	Pain	1 (0.8)		—	
	Eyesight	1 (0.8)		—	

^a^ECG: electrocardiogram.

^b^Not available.

#### Area 3: Older Adults’ Relationship With Technology

Multimedia Appendix 2 provides the results from the *web-based questionnaire for older adults* related to *older adults’ relationship with technology*.

#### Area 4: Use of Technology in Medical Practice

Table 5 illustrates the clinicians’ results (*focus group for clinicians and web-based questionnaire for clinicians*) related to the *use of technology in medical practice*.

As reported in Table 5, smart devices were considered useful in medical practice by most participants (107/118, 90.7%). However, 42.4% (50/118) of the clinicians never used smart devices in clinical practice, whereas only 5.1% (6/118) used them all the time. The same percentage of participants (50/118, 42.4%) did not suggest any of the proposed or other devices, whereas 1.7% (2/118) of clinicians judged the use of a device to recognize the patient’s position and movement (eg, GPS tracking device) as significant. Monitoring of a person’s sleep quality and oxygen saturation was also suggested by a participant.

**Table 5:** Results related to the use of technology in medical practice (N=118).

Use of technology		Clinicians, n (%)
Technology is useful in medical practice		107 (90.7)
**Frequency of use**		
	Never	50 (42.4)
	Rarely	34 (28.8)
	Sometimes	14 (11.9)
	Often	14 (11.9)
	Everyday	6 (5.1)
**Apps and devices prescribed or suggested**		
	Nutrition app	20 (16.9)
	Physiotherapy app and smart devices	23 (19.5)
	Smart hearing aids	10 (8.5)
	Smart pillboxes	23 (19.5)
	Physical activity app and smart devices	23 (19.5)
	Smart blood pressure tracker	32 (27.1)
	None	50 (42.4)
	Other: movement tracking	2 (1.7)
	Other: oxygen saturation and sleep quality app	1 (0.8)

#### Area 5: About SMART BEAR

Most clinicians (108/118, 91.5%) would recommend their patients to participate in the SMART BEAR project. Moreover, almost all participants would like to receive regular reports regarding the patients’ health status. More specifically, they preferred daily reports (8/118, 6.7%), weekly reports (58/118, 49.1%), and monthly reports (35/118, 29.6%). Approximately 11.8% (14/118) of the participants would like to receive a report only if an abnormality was detected, whereas 1.7% (2/118) of the participants did not answer. During the *focus group for clinicians*, 94.1% (111/118) of the clinicians expressed interest in sending the reports to the patient, 75.4% (89/118) of them suggested sending the reports to both physicians and patients, and 38.1% (45/118) suggested sending the reports to the patient and caregiver.

Even older adults (18/24, 75%) expressed interest in receiving periodic reports about their own health status (weekly reports were preferred by 4/24, 17%, whereas monthly reports were favored by 20/24, 83%). In addition, they would like their own clinician (8/24, 33%), wife or husband (9/24, 38%), son or daughter (7/24, 29%), or none (4/24, 17%) to have access to the content of those reports. Regarding how to receive the reports, written messages (17/24, 71%), voice messaging (3/24, 13%), and email (9/24, 38%) were indicated. Approximately 63% (15/24) of the older adults showed an interest in notifications and suggestions that the platform could generate based on the collected data. This information was expected to be shared with the spouse (11/24, 46%), son or daughter (6/24, 25%), physician (2/24, 8%), and friend (2/24, 8%), whereas some (3/24, 13%) of participants preferred that no one had access to such notifications and suggestions. The expressed preferred ways of receiving notifications and suggestions were written messages (18/24, 75%), voice messaging (2/24, 8%), and email (10/24, 42%).

Table 6 reports the main clinicians’ (*focus group for clinicians and web-based questionnaire for clinicians*) and older adults’ (*web-based questionnaire for older adults*) inputs related to about SMART BEAR.

At the end of the *requirements collection* phase, the outcomes gained by considering both clinicians’ and older adults’ opinions and suggestions were mapped to the domains of the IC model to define a list of interventions and transversal functions worth including in the platform.

**Table 6:** Results related to about SMART BEAR (N=142).

About SMART BEAR				Clinicians (n=118), n (%)		Older adults (n=24), n (%)
Participation in SMART BEAR project				108 (91.5)		16 (66.7)
**Expectations**						
	Less unnecessary visits		40 (33.9)		3 (12.5)	
	Enhanced patient’s safety		39 (33.1)		9 (37.5)	
	Better self-management of patients’ health status		77 (65.3)		10 (41.7)	
	Better patient’s social interactions		37 (31.4)		4 (16.7)	
	None		2 (1.7)		3 (12.5)	
	No answer		2 (1.7)		—^a^	
	**Other**					
		Enhanced patient’s autonomy	—		3 (12.5)	
		Enhanced patient’s confidence	55 (46.6)		4 (16.7)	
		Improved patient’s health status	12 (10.2)		—	
		Improved patient’s diet habits	28 (23.7)		2 (8.3)	
		Improved patient-physician communication	9 (7.6)		—	
		Time saving	23 (19.5)		6 (25)	
		Money saving	19 (16.1)		2 (8.3)	
**Concerns**						
	Privacy		19 (16.1)		5 (20.8)	
	Change of routine		28 (23.7)		3 (12.5)	
	Erroneous measurements		37 (31.4)		4 (16.7)	
	Erroneous notifications (suggestions by platform)		31 (26.3)		3 (12.5)	
	Technical issues of the devices		60 (50.8)		7 (29.2)	
	Education on devices and platform use		75 (63.6)		2 (8.3)	
	Increased stress for the user		25 (21.2)		5 (20.8)	
	None		4 (3.4)		6 (25)	
	No answer		1 (0.8)		—	
	Other: decreased patient’s referral to private practice		2 (1.7)		—	

^a^Not available.

### Evaluation Phase

A total of 6 participants took part in *the focus group for older adults* (5/6, 83% women) in the *evaluation* phase. One of the participants was aged <70 years, whereas the others were in the 71 to 75 age range group. The education of this sample was heterogeneously distributed (ie, 1/6, 17% elementary school; 2/6, 33% middle school; 1/6, 17% high school; and 2/6, 33% in university). Half of the sample declared that they lived alone, whereas the other half stated that they lived with their spouse. Regarding the medical conditions experienced by participants, they claimed to have experienced hypertension (6/6, 100%), anxiety (3/6, 50%), cardiovascular diseases (1/6, 17%), and hearing loss (1/6, 17%).

The results of the assessment of the SMART BEAR platform interventions and transversal functions are reported in Table 7.

Overall, participants evaluated the platform positively, and all participants agreed that the objectives would be achieved. In particular, in their opinion, the SMART BEAR platform would enhance self-awareness of users’ health status (6/6, 100%), support self-management of users’ health conditions (6/6, 100%), promote active living both physically and cognitively (6/6, 100%), facilitate independent living (5/6, 83%), and enable evidence-based support for clinicians (6/6, 100%).

**Table 7:** Older users’ assessment of SMART BEAR functions in the evaluation phase (N=6).

Functions of SMART BEAR platform			Usefulness, n (%)		Credibility, n (%)		Desirability, n (%)		Learnability, n (%)
**Interventions**									
	Physical training	6 (100)		6 (100)		5 (83)		2 (33)	
	Diet plan	5 (83)		4 (67)		1 (17)		2 (33)	
	Monitoring of physiological parameters	6 (100)		6 (100)		6 (100)		5 (83)	
	Psychoeducational intervention	6 (100)		6 (100)		4 (67)		2 (33)	
	Monitoring of the mood	4 (67)		4 (67)		3 (50)		3 (50)	
	Cognitive training	6 (100)		6 (100)		6 (100)		6 (100)	
	Hearing training	6 (100)		6 (100)		6 (100)		6 (100)	
	Environment monitoring and adjustment	1 (17)		1 (17)		0 (0)		0 (0)	
**Transversal functions**									
	Data visualization	6 (100)		N/A^a^		5 (83)		N/A	
	Gamification	6 (100)		N/A		5 (83)		N/A	
	Regular reports	6 (100)		N/A		5 (83)		N/A	
	Regular report to clinician	4 (67)		N/A		5 (83)		N/A	
	Suggestion	6 (100)		N/A		6 (100)		N/A	
	Reminder	6 (100)		N/A		6 (100)		N/A	
	Data access to caregiver	2 (33)		N/A		2 (33)		N/A	
	Teleconsulting	6 (100)		N/A		5 (83)		N/A	

^a^N/A: not applicable.

## Discussion

### Principal Findings

The goal of this study was 2-fold; we aimed to understand stakeholders’ beliefs, attitudes, needs, expectations, and concerns about the SMART BEAR platform (*objective 1*) and to evaluate the proposed solution (*objective 2*) with a small group of older adults.

Regarding *objective 1*, a thorough comprehension of clinicians’ and older adults’ perceptions of the SMART BEAR platform was pursued through the investigation of five areas: *impact of disease in everyday life* (area 1), *remote monitoring* (area 2), *older adults’ relationship with the technology* (area 3), *use of technology in medical practice* (area 4), and *about SMART BEAR* (area 5).

### Impact of Disease in Everyday Life

The results obtained in the *requirements collection* phase showed that clinicians agreed on the impact that age-related conditions have on older adults’ daily living activities, which was also confirmed by the interviewed older adults. More specifically, the effects on all the areas investigated within the IC model were reported by both clinicians and older participants: psychological area (13/16, 81% clinicians and 17/24, 71% older adults), vital area (15/16, 94% clinicians and 13/24, 54% older adults), motor area (9/16, 56% clinicians and 14/24, 58% older adults), cognitive area (10/16, 63% clinicians and 10/24, 42% older adults), and sensorial area (6/16, 38% clinicians and 11/24, 46% older adults). More specifically, older adults complained of anxiety, irritability, reduced energy, problems in vision, hearing, memory, attention difficulties, cardiorespiratory problems, and sleep disorders. However, the older adults who answered the questionnaire did not acknowledge the impact of the age-related conditions on their vital areas as severely as the interviewed clinicians assessed their patients. This might be because of a more objective overview of the vital problems from the clinicians (ie, objective medical examination and appropriate measurement of vital signs) than the subjective older adults’ self-awareness. Although input from clinicians is fundamental to the design of an assistive technology, this discrepancy supports the importance of tailoring the smart health platform to the needs of a specific patient. In fact, as it emerged from the literature review, one of the most frequent causes of technology abandonment in older adults is the lack of perceived relevance of the service [23].

The onset of chronic diseases in the older population has important consequences for family members as well. Indeed, clinicians (ie, 14/16, 88% of participants in the *focus group for clinicians*) underlined that the complex management of the patient (eg, daily assistance and medical appointments) might lead caregivers to mental health problems (eg, sleeping difficulties, burnout, and depression), as confirmed by the literature [31,32]. Nevertheless, only 29% (7/24) of the older adults in *web-based questionnaire for older adults* declared to need help from family members for routine activities (eg, personal hygiene and visits to the physician). This incomplete overlap between clinicians’ and older adults’ feedback is in line with the relatively low limitations that older respondents have reported on their autonomy. This may be explained by the age of the sample population (20/24, 83% of older adults were aged <76 years, whereas more severe comorbidities were generally observed in more advanced age). The age distribution of older participants, in turn, could be because of the method used for data collection (ie, web-based questionnaire), which requires a certain autonomy and familiarity with the technology.

### Use of Technology in Medical Practice

The outpatient approach for age-related disorders is the most preferred and used by interviewed clinicians. This is probably why most of them (107/118, 90.7%) considered the technology useful in medical practice (Table 5). These results are in line with those of the current literature [33,34]. In particular, objective measurements provided by smart medical devices are very appealing for medical experts as they can ensure support in decision-making for the clinician, help for the caregiver, and timely interventions for the patient. This study suggests that the most used devices in clinical practice are smart blood pressure trackers (32/118, 27.1%), physical activity and physiotherapy applications or devices (23/118, 19.5%), smart pillboxes (23, 19.5%), and nutrition applications (20/118, 16.9%). Furthermore, applications that are able to monitor sleep quality and track position were also indicated to cope with older adults’ sleeping difficulties and cognitive problems. Instead, smart hearing aids were suggested or prescribed by only 8.5% (10/118) of clinicians, and such data might be due to the expertise of the clinicians involved in this study (ie, only a few of them, 11/118, 9.3%, deal with hearing impairments; Table 2). However, the use of smart devices in the current clinical practice is still uncommon (50/118, 42.4% of clinicians never use smart devices; Table 5), and it is mostly explained by the difficulties met by the older population in technology adoption [21,25].

### Older Adults’ Relationship With Technology

Older adults who took part in the study suggested being quite confident with the technology use (Multimedia Appendix 2). Only 8% (2/24) of the participants found some difficulties in using it, and none judged it as obstructive to everyday life. However, it is important to note that although the totality of participants regularly uses a smartphone, a decreasing trend in the use of more modern devices can be observed (ie, 15/24, 63% uses smart television, 9/24, 38% uses smartwatches, and 6/24, 25% uses smart lamps and smart thermostats). Nevertheless, this apparent resistance to the latest generation of devices can be overcome, provided that the technology is found valuable. Indeed, most participants were positively predisposed to use new technology if considered helpful, and none expressed themselves against adopting a useful device. Such findings are also confirmed by Vaportzis et al [21].

### Remote Monitoring

Both clinicians and older adults agreed on the importance of remote monitoring. However, greater participation in identifying parameters useful for remote monitoring was observed from clinicians rather than older adults (ie, 4/244, 1.6% of older participants did not express any suggestions about measurement to monitor remotely; Table 4). The interest of health care professionals in monitoring blood pressure (85/118, 72%), heart rate (65/118, 55.1%), and diet habits (68/118, 57.6%), as well as physical activity and sleep quality, are in line with the typologies of devices and applications that they recommend to patients in their clinical practice. Further interest was also shown in devices that may detect falls (74/118, 62.7%) and monitor patients’ therapy adherence and mood, cognitive functions, and behavioral changes of the latter. In this way, it is indeed possible to increase older patients’ safety, provide concrete support for their caregivers in the management of the therapy, and help older adults face loneliness and social exclusion. Even older adults considered blood pressure (12/24, 50%), diet habits (8/24, 33%), and heart rate (5/24, 21%) measurements to be relevant. On the other hand, fall detection was not considered crucial (it was judged useful for 2/24, 8% of older respondents), which may be explained by the low rate of motor problems in the older adults’ sample (Table 2). Similarly, social contact frequency tracing aroused little interest in older adults (2/24, 8%) as it felt intrusive. Clinicians registered some concerns about remote monitoring; they complained about the shortage of time to answer patients’ phone calls and lack direct interaction with the patient. In addition, they stated that the symptoms remotely reported by patients could be misleading (ie, subjective) and hence misinterpreted in the absence of a visit in person. Finally, they said they were worried about potential false positives (ie, receiving alarm values from the device that could be normal for a specific patient).

### About SMART BEAR

Smart health platforms, such as SMART BEAR, have attracted interest from both stakeholders. Most of them (108/118, 91.5% of clinicians and 16/24, 67% of older adults) expressed interest in participating in the project. The main expectation from using the SMART BEAR platform is a better self-management of patients’ health status (77/118, 65.3% of clinicians and 10/24, 42% of older adults; Table 6). Both clinicians and older adults were aligned with the expectation that the SMART BEAR platform may increase patient safety (39/118, 33.1% of clinicians and 9/24, 38% of older adults). In contrast, it appears that only clinicians expected that this platform might reduce patients’ unnecessary visits (40/118, 33.9% of clinicians and 3/24, 13% of older adults) and increase patients’ social interactions (37/118, 31.4% for clinicians and 4/24, 17% for older adults). Clinicians also awaited enhanced patient confidence and improved patient-physician communication. However, the latter seems to be more concerned with the use of such a platform than older adults (ie, 4/118, 3.4% of clinicians vs 6/24, 25% of older adults declared to have none of the proposed concerns). Moreover, a difference in the typology of concerns reported by stakeholders was observed (Table 6). For example, the health care professionals’ sample was especially concerned with education on devices and platform use (75/118, 63.6%), which is in contrast observed for only a few (2/24, 8%) of the older adults. In fact, no particular criticalities in using the technology were reported by the older people involved in the study, which may be because of the age distribution of the sample or participants’ underestimation of the difficulties in using technological devices. Conversely, poor usability and improper functioning of the platform (eg, technical issues of devices, erroneous measurements, and notification and suggestion by the platform) caused concerns for both stakeholders. Furthermore, a small group of both clinicians and older adults (ie, 25/118, 21.2% of clinicians and 5/24, 21% of older adults) reported that the use of the proposed technology could lead to increased stress for the users. In particular, older adults (ie, 5/24, 21% of older adults vs 19/118, 16.1% of clinicians) were worried about privacy issues, which is fully understandable as it is subject to monitoring provided by the platform [20,21].

With regard to objective 2, the usefulness, credibility, desirability, and learnability of the SMART BEAR functions for its end users were assessed. The results obtained in the evaluation phase showed that the presented technology could address all the expected goals. Moreover, most of the proposed interventions were well-accepted by older adults. In more detail, all participants evaluated cognitive training (intervention 6) and hearing training (intervention 7) as useful, credible, desirable, and easy to learn. A very positive assessment of usefulness and credibility was also gained for physical training (intervention 1), monitoring of physiological parameters (intervention 3), and psychoeducational intervention (intervention 4). However, some doubts about the ease of use of such functionalities, with special regard to physical training and psychoeducational intervention, were revealed by older adults. The possibility of monitoring diet habits (intervention 2) was found useful by most people (5/6, 83%) but not as attractive as it was judged invasive (it was considered desirable only for the 1/6, 17%; Table 7).

In contrast, almost all participants who judged the monitoring of mood as useful (intervention 5) would like to have it (ie, 4/6, 67% and 3/6, 50%, respectively; Table 7). Such functionality has been judged effective in gaining a greater awareness of the patient’s own condition. Nonetheless, uncertainty about a computer-based interaction on emotions and states of mind was raised (eg, “describing my mood using a smartphone without a person to person communication is unfriendly”). In addition, participants reported concerns that such an intervention (ie, intervention 5) may be time consuming and too burdensome (eg, to fill in a web-based questionnaire weekly). The environment monitoring and adjustment (intervention 8) received a negative assessment; indeed, only 17% (1/6) considered it valid and credible, and none would like to use it (eg, “it is difficult to accept a change in the own routine when the age is advancing” and “it is challenging to find the environmental conditions that fit well with all family members”). However, such functionality (ie, intervention 8) was considered helpful for less autonomous individuals. Relative to transversal functions, they were believed advantageous and desirable, although some preferences could be observed. The idea of receiving notifications and suggestions (transversal function 5) and the reminders (transversal function 6) from the platform were highly appreciated by all older users. Furthermore, the possibility of visualizing data (transversal function 1), access gamification dynamics (transversal function 2) for an enhanced motivation in pursuing the program, get information (eg, trend and statistics) about their own behaviors and health status through regular reports (transversal function 3), and seek a medical teleconsultation using the platform (transversal function 8) were thought helpful by everyone but undesirable by one of the participants because of poor confidence in the technology. Sharing regular reports with clinicians (transversal function 4) was found desirable by 83% (5/6) of participants but useful by 67% (4/6) of participants as they had some doubts that the clinician would agree to use this feature (ie, it takes considerable time). Finally, the possibility of sharing the data collected by the platform about their health parameters, activities, and behaviors with caregivers (transversal function 7) was widely discussed among older adults, and the willingness to safeguard their privacy and to feel independent but also the desire not to worry their loved ones and a light embarrassment in using gerontological technologies were reported. All these factors led most older participants (4/6, 67%) to consider data access to caregivers useless and undesirable.

### Conclusions, Limitations, Strengths, and Future Works

In the era of personalized medicine, several benefits are expected from innovative smart health technologies that are able to ensure a continuous and noninvasive remote monitoring of the patient. For example, an early diagnosis; a data-driven approach in medical assistance; a closer and more trustful physician–patient relationship; and improved self-management, autonomy, and safety of the patient are desired.

In this study, clinicians’ and older adults’ perspectives about a smart health platform were gathered to design a solution (ie, the SMART BEAR platform) that fits all end user requirements well. The obtained results showed that the SMART BEAR platform represents a suitable solution for improving older adults’ QoL, reducing the burden of age-related chronic conditions for both patients and caregivers, and providing objective reporting to the clinician for evidence-based medicine. Moreover, it offers useful insights so that smart health can become a widespread reality. For instance, devices and applications specifically targeted for the older population should not contain stigmatizing symbols, thus avoiding negative feelings in older adults with a consequent failed adoption of the technology. In addition, users’ needs and expectations to meet and concerns to address and solve were defined. For example, the service needs to offer adequate training and technical support for end users to be endorsed by clinicians. Finally, several functionalities for a successful smart health platform were suggested, such as psychoeducational interventions and gamification elements.

This study used a mixed approach, adopting qualitative (ie, focus groups) and quantitative methods (ie, questionnaires with close-ended questions). This approach has limitations, as it does not allow a complete comparison between the data obtained with different methods; however, it is most effective for gaining insights into the issue. In fact, focus groups enable open discussions in which researchers can explore a subject with experts. Conversely, web-based questionnaires are a powerful method for maximizing participation in an investigation, thus consolidating or disproving assumptions formulated during the first exploratory phase. The following collection of technology requirements was based on data from a large sample. The designed platform has features that appeal to at least a consistent group of experts and a smaller group of possible users. Ultimately, focus groups become useful again to validate the elaborated concept and gain a detailed impression from potential end users. However, the sample of older adults was limited in comparison with the sample of clinicians, and this may be a limitation that can hardly be overcome because of the difficulty in reaching that population in large numbers. Moreover, it should be noted that the obtained outcomes could have been affected by the characteristics of the participants involved (ie, age groups and age-related conditions were not equally covered by participants).

Regarding research positioning, the authors mainly faced cultural challenges because of demographic differences between the research group and the group under study; that is, older adults. Geriatric medical specialists were essential to adopt a fitting framework for understanding the problem and the objectives of this research. This allowed researchers to design the study methodology and tools by adopting a holistic, person-centered approach instead of the traditional disease-based approach. This point of view was supported by the technical–biological background of the authors with a biomedical engineering degree, especially in studying how novel technological interventions would be able to support the individual’s well-being. As this study involved the active participation of older adults, the researchers tried to consider possible biases when the surveys were designed and conducted. This aspect was particularly important for the validation of the proposed design in focus groups when technology was discussed with participants who probably had very limited experience with it. Hence, the mediation of a neuropsychologist ensured that a common understanding was created with the participants and that possible adverse outcomes such as misunderstandings and frustration were avoided. Overall, heterogeneity in the academic background was the key strength of the research group.

Future works comprising further experimental activities with more and varied stakeholders (ie, clinicians and older adults distributed heterogeneously concerning the age, sex, and medical conditions treated or experienced and also their caregivers) are needed to investigate the acceptability and the overall user experience of the future developed platform in free-living conditions.

## Appendix

Multimedia Appendix 1 Material used in both the requirements collection phase and the evaluation phase.

Multimedia Appendix 2 Results from the web-based questionnaire for older adults related to older adults’ relationship with technology.

## Abbreviations

IC: intrinsic capacity
IRCCS: Istituto di Ricovero e Cura a Carattere Scientifico
QoL: quality of life
