# Supplementary material for: Older Adults’ and Clinicians’ Perspectives on a Smart Health Platform for the Aging Population: Design and Evaluation Study
Source: JMIR Aging. 2022 Feb 28;5(1):e29623. doi: 10.2196/29623 (PMC8922154; doi:10.2196/29623)
Supplement: Multimedia Appendix 1 [file aging_v5i1e29623_app1.docx]

**Multimedia Appendix 1**

***SMARTBEAR FOCUS GROUP CLINICIANS***

## QUESTIONNAIRE

### Demographic Data

**Q1.** Age ………….………………………………………………………………………………………………………………………

**Q2.** Gender M ⃝ F ⃝

**Q3.** Occupation ……………………………..………………………………………………………………………………………………..

**Q4.** Do you treat any of the following conditions?

*Please check (*✓*) all that apply for you and feel free to add any further comments*

| *Hearing loss* | *⃝* | *Dementia* | *⃝* |
| --- | --- | --- | --- |
| *Imbalance* | *⃝* | *Falls* | *⃝* |
| *Anxiety* | *⃝* | *Stress* | *⃝* |
| *High blood pressure* | *⃝* | *Depression* | *⃝* |
| *Ischemic heart disease* | *⃝* | *Arrhythmias* | *⃝* |

**Q5.** Experience

*Years spent treating this medical condition* …………………………………………………………………………………….

**Q6.** Does this medical condition require for a personalized treatment? If it does, what should a personalized treatment plan include?

### A1 – Impact of the disease in everyday life

*[Investigate eventual difficulties /complications/challenges for the patient and his/her caregivers]*

- 1. What is the impact of this medical condition on the patients’ activities of daily living? Which are the main issues encountered by the patient at home?

*For example, does it affect daily life activities, nutrition, sleep, mood, motor capacity, cognition (e.g. memory, attention and language)?*

| ***Vitality***  (daily life activities, cardio-respiratory functions, nutrition, etc..) | ***_*** |
| --- | --- |
| ***Sensory***  (vision, hearing, etc..) | ***_*** |
| ***Psychological***  (mood, sleep, etc) | ***_*** |
| ***Locomotion***  (balance, motor capacity, gait, etc..) | ***_*** |
| ***Cognition***  (memory, attention, language, etc..) | ***_*** |

- 1. Who are the people directly or indirectly affected by the onset of the medical condition? (E.g., family members, relatives, health care providers)
  2. How do these difficulties affect the caregiver’s life? Which are the main issues encountered by the caregivers?
  3. Which are the cons of the current medical procedure for the patient? (E.g., remind to take the drugs, handling the adverse effects related to the medication, etc.)
  4. Which are the cons of the current medical procedure for the people close to the patient? (E.g., anxiety about continuous monitoring, remind about the clinical appointments etc.)

### A2 – Remote monitoring

*[Explore remote monitoring]*

- 1. Remote monitoring

*Have you ever been dealing with remote monitoring technology?*

*Yes ⃝ No ⃝*

- 1. Which are the pros and cons of the current medical procedure for the clinician? (E.g., use of WhatsApp for instant communication considered as pro but the technology invasiveness affects private life, etc.)
  2. What kind of treatment is used for this condition? What is the mostly used treatment (e.g. inpatient or outpatient treatment)?
  3. How many patients do you monitor outside of conventional clinical settings? (e.g. such as in the home or in a remote area, etc.).

*Please indicate the percentage of patients ………..…………………………………………………………………………..*

- 1. What do you usually monitor outside your clinical setting? (E.g., pharmacological therapy, cognitive, organic or mood symptoms, etc.)
  2. How do you communicate remotely with the patient? Please indicate what kind of technology or platforms you use. (E.g., whatsApp, internet platforms, phone calls…)
  3. Does this remote monitoring cause burden on you? (For example in terms of stress, time…)
  4. How would you improve weakness/cons of the current medical procedure?
  5. Which of the following measurements would you consider as more useful to your patients’ everyday life?

*Please check (*✓*) up to 3 boxes and feel free to add any further comments*

| Blood pressure | ⃝ | Heart rate | ⃝ |
| --- | --- | --- | --- |
| House temperature | ⃝ | Blood sugar | ⃝ |
| Air pollution | ⃝ | Social interaction frequency | ⃝ |
| Electrocardiogram | ⃝ | Dietary habits | ⃝ |
| Fall detection | ⃝ | Levels of noise exposure | ⃝ |

- 1. What would be the benefit in remote patient monitoring for 24 hours a day, if any?

### A4 – Use of technology in medical practice

*[Explore the use of technology in medical practice]*

- 1. Which of the following devices have you used/ prescribed/ suggested to your patients? Please check (✓) all that apply and feel free to add any further comments

| *Nutrition Applications* | *⃝* | *Smart pillboxes* | *⃝* |
| --- | --- | --- | --- |
| *Physiotherapy Applications and Smart Devices* | *⃝* | *Physical Activity Applications and Smart Devices* | *⃝* |
| *Smart Hearing Aids* | *⃝* | *Smart Blood Pressure Tracker* | *⃝* |

- 1. How often do you use Smart Devices in your everyday clinical practice?

*Please check (*✓*) only one box and feel free to add any further comments*

| *Never* | *Rarely* | *Sometimes* | *Often* | *Always* |
| --- | --- | --- | --- | --- |
|  |  |  |  |  |

- 1. How useful do you find technology for your clinical practice?

*Please check (*✓*) only one box and feel free to add any further comments*

| *Obstructive* | *Indifferent* | *Useful* | *Very useful* | *Fundamental* |
| --- | --- | --- | --- | --- |
|  |  |  |  |  |

- 1. How easy do you find technology to use in your clinical practice?

*Please check (*✓*) only one box and feel free to add any further comments*

| *Impossible* | *Difficult* | *Neutral* | *Easy* | *Very easy* |
| --- | --- | --- | --- | --- |
|  |  |  |  |  |

### A5 – About an eventual experience with SMARTBEAR

- 1. **SMARTBEAR** Platform could provide you with regular reports concerning your patients’ health status and measurements. Is that something appealing to you?

*Please check (*✓*) only one box and feel free to add any further comments*

Yes ⃝ No ⃝

- 1. **SMARTBEAR** Platform could provide you with regular reports concerning your patients’ health status and measurements. Which type of report would you consider as more helpful to them and to you?

*Please check (*✓*) only one box and feel free to add any further comments*

Monthly report ⃝ Weekly report ⃝ Daily report ⃝

- 1. How would you like to receive this information? (E.g., background message, text messages, email, etc.)
  2. **SMARTBEAR** Platform could provide patients with regular notifications – suggestions depending on its observations e.g. “Your blood pressure is regularly higher than the normal for the last 2 weeks. You should visit your referring physician”.

Which type of notifications would you consider as more helpful?

*Please check (*✓*) only one box and feel free to add any further comments*

Notifications to the user ⃝

Notifications to the user and his/her referring physician* ⃝ Notifications to the user and his/her significant other* ⃝

* (with user’s consent)

- 1. How would you like to receive these regular notifications – suggestions? (E.g., background message, text message, email, etc.)
  2. Would you encourage your patients to participate in a project implementing state of the art technology to their everyday life, such as **SMARTBEAR**? Why?

*Please check (*✓*) only one box and feel free to add any further comments*

Yes ⃝ No ⃝

- 1. What are your expectations of a project like **SMARTBEAR**?

*Please check (*✓*) up to 3 boxes and feel free to add any further comments*

| *Less unnecessary visits* | *⃝* |
| --- | --- |
| *Frail Patients’ Safety* | *⃝* |
| *Better auto-management of your patients’ health issues* | *⃝* |
| *Better social interactions* | *⃝* |
| *Improving of your patients’ overall health* | *⃝* |
| *Improving your patient – doctor communication* | *⃝* |

- 1. If one of your patients asks for your opinion on whether to participate in **SMARTBEAR** Project, which would be your major concerns?

*Please check (*✓*) up to 3 boxes and feel free to add any further comments*

| *Privacy* | *⃝* |
| --- | --- |
| *Change of Routine* | *⃝* |
| *Erroneous Measurements* | *⃝* |
| *Erroneous Notifications – Suggestions by the Platform* | *⃝* |
| *Technical Issues of the Devices – Internet Connection* | *⃝* |
| *Education on Devices and Platform Usage* | *⃝* |
| *Increased stress for the user* | *⃝* |

**Would you like to add any further comments? If yes, please add your comments to the box below.**

**Thank you for taking the time to fill our questionnaire!**

***SMARTBEAR ONLINE QUESTIONNAIRE CLINICIANS***

## QUESTIONNAIRE

### Demographic data

**Q1.** What is your medical domain of expertise?

*Please choose only one of the following:*

| *ENT* | *⃝* |
| --- | --- |
| *Cardiology* | *⃝* |
| *General Medicine* | *⃝* |
| *Geriatrics* | *⃝* |
| *Neurology* | *⃝* |
| *Psychiatry* | *⃝* |
| *Other* | *⃝* |

**Q2.** Do you treat any of the following conditions?

*Please choose all that apply:*

| *Hearing loss - Tinnitus* | ⃝ | *Ischemic heart disease* | ⃝ |
| --- | --- | --- | --- |
| *Falls* | ⃝ | *Hypertension* | ⃝ |
| *Imbalance* | ⃝ | *Dementia* | ⃝ |
| *Arrhythmias* | ⃝ | *Anxiety* | ⃝ |
| *Stress* | ⃝ | *Depression* | ⃝ |

### A2 – Remote monitoring

*[Explore remote monitoring]*

- 1. How often does your average aged patient visits you?

*Please choose only one of the following:*

| *More than once per month* | *⃝* |
| --- | --- |
| *Once per 1-3 months* | *⃝* |
| *Once per 6 months* | *⃝* |
| *Once per year* | *⃝* |

- 1. Which of the following measurements would you consider more useful for your patients’ everyday wellbeing?

*Please choose all that apply:*

| *Blood pressure* | ⃝ | *Heart rate* | ⃝ |
| --- | --- | --- | --- |
| *House temperature* | ⃝ | *Blood sugar* | ⃝ |
| *Air pollution* | ⃝ | *Social interaction frequency* | ⃝ |
| *Electrocardiogram* | ⃝ | *Dietary habits* | ⃝ |
| *Fall detection* | ⃝ | *Levels of noise exposure* | ⃝ |
| *Cognitive decline* | ⃝ | *Emotional Changes* | ⃝ |

### A4 – Use of technology in medical practice

- 1. Which of the following devices have you used/ prescribed/ suggested to your patients?

*Please choose all that apply:*

| *Nutrition Applications* | *⃝* | *Smart pillboxes* | *⃝* |
| --- | --- | --- | --- |
| *Physiotherapy Applications and Smart Devices* | *⃝* | *Physical Activity Applications and Smart Devices* | *⃝* |
| *Smart Hearing Aids* | *⃝* | *Smart Blood Pressure Tracker* | *⃝* |

- 1. How often do you use Smart Devices such as Smart blood pressure tracker or Smart watches in your everyday clinical practice?

*Please choose only one of the following:*

| *Never* | *⃝* |
| --- | --- |
| *Rarely* | *⃝* |
| *Sometimes* | *⃝* |
| *Often* | *⃝* |
| *Everyday* | *⃝* |

- 1. Do you think that implementing state of the art technology to your aged patients’ everyday life would be overall helpful?

*Please choose only one of the following:*

Yes ⃝ No ⃝

### A5 – About SMART BEAR Project

- 1. Would you recommend your patients to participate in a project implementing state of the art technology to their everyday life, such as SMART BEAR?

*Please choose only one of the following:*

Yes ⃝ No ⃝

- 1. Which are your major concerns about SMART BEAR project?

*Please choose all that apply:*

| *Privacy* | *⃝* |
| --- | --- |
| *Change of Routine* | *⃝* |
| *Erroneous Measurements* | *⃝* |
| *Erroneous Interventions – Suggestions by the Platform* | *⃝* |
| *Technical Issues of the Devices – Internet Connection* | *⃝* |
| *Education on Devices and Platform Usage* | *⃝* |
| *Increased stress for the user* | *⃝* |
| *Decreased patients’ referral to your private practice* | *⃝* |

- 1. Which are your expectations about your patients’ participation in SMART BEAR project?

*Please choose all that apply:*

| *Less unnecessary visits* | *⃝* |
| --- | --- |
| *Safety* | *⃝* |
| *Better self-management of patients’ health issues* | *⃝* |

| *Improved social interactions* | *⃝* |
| --- | --- |
| *Time Saving* | *⃝* |
| *Money Saving* | *⃝* |
| *Improving their diet habits* | *⃝* |
| *Improving their confidence - independence* | *⃝* |

- 1. SMART BEAR Platform could provide participants and their referring physicians with regular reports concerning their health status and measurements. How often do you think the system should generate these reports?

*Please choose only one of the following:*

| *Daily* | *⃝* |
| --- | --- |
| *Weekly* | *⃝* |
| *Monthly* | *⃝* |
| *Only if an abnormality is detected* | *⃝* |

- 1. Do you think that these reports would be overall helpful for your patients?

*Please choose only one of the following:*

Yes ⃝ No ⃝

- 1. SMART BEAR platform could provide referring physicians with access to their patients’ data and ask for their input after each patient’s visit. Is that something appealing to you?

*Please choose only one of the following:*

Yes ⃝ No ⃝

**Thank you for taking the time to fill our questionnaire!**

***SMARTBEAR ONLINE QUESTIONNAIRE OLDER ADULTS***

## QUESTIONNAIRE

### Demographic data

**Q1. Age group**

| *65-70 y.o.* | *⃝* |
| --- | --- |
| *71-75 y.o.* | *⃝* |
| *76-80 y.o.* | *⃝* |
| *81+ y.o.* | *⃝* |

**Q2. Gender** M ⃝ F ⃝

**Q3. Education** ……………………………..………………………………………………………………………………………………..

**Q4. Do you suffer from any of the following conditions?**

*Please check (*✓*) all that apply for you and feel free to add any further comments*

| *Hearing loss* | *⃝* | *Dementia* | *⃝* |
| --- | --- | --- | --- |
| *Imbalance* | *⃝* | *Falls* | *⃝* |
| *Anxiety* | *⃝* | *Stress* | *⃝* |
| *High blood pressure* | *⃝* | *Depression* | *⃝* |
| *Ischemic heart disease* | *⃝* | *Arrhythmias/Cardiovascular disease* | *⃝* |

**Q5. Do you live with someone?**

Yes ⃝ No ⃝

**Q6. If yes, please indicate who you live with**

| *Partner/Spouse* | ⃝ | *Caregiver* | ⃝ |
| --- | --- | --- | --- |
| *Son/daughter* | ⃝ | *Other* | ⃝ |

## A1 – Impact of the disease in everyday life

- 1. **What is the impact of your medical condition on your vital area?**

| ▢ | *Cardio-respiratory functions* |
| --- | --- |
| ▢ | *Diet* |
| ▢ | *Autonomy* |
| ▢ | *Sleep* |
| ▢ | *None of the above* |

- 1. **What is the impact of your medical condition on your sensorial area?**

| ▢ | *Eyesight* |
| --- | --- |
| ▢ | *Hearing* |
| ▢ | *Touch* |
| ▢ | *Smell* |
| ▢ | *Taste* |
| ▢ | *None of the above* |

- 1. **What is the impact of your medical condition on your psychological area?**

| ▢ | *Anxiety* |
| --- | --- |
| ▢ | *Depression* |
| ▢ | *Euphoria* |
| ▢ | *Irritability* |
| ▢ | *Energy* |
| ▢ | *Sociality* |
| ▢ | *None of the above* |

- 1. **What is the impact of your medical condition on your motor area?**

| ▢ | *Balance* |
| --- | --- |
| ▢ | *Locomotion* |
| ▢ | *Coordination* |
| ▢ | *Muscle strength* |
| ▢ | *None of the above* |

**1.5 What is the impact of your medical condition on your cognitive area?**

| ▢ | *Memory* |
| --- | --- |
| ▢ | *Attention* |
| ▢ | *Language* |
| ▢ | *None of the above* |

- 1. **How autonomous are you in handling your medical condition?**

| ⃝ | I regularly need help |
| --- | --- |
| ⃝ | I sometimes need help |
| ⃝ | I rarely need help |
| ⃝ | I don’t need any help |

- 1. **In which daily activity do you need help or assistance?**

| ▢ | Grocery shopping |
| --- | --- |
| ▢ | Going for walks |
| ▢ | Personal hygiene |
| ▢ | Going to the doctor |
| ▢ | Cooking |
| ▢ | None of the above |
| ▢ | Other |

## A2 - Remote Monitoring

- 1. **How often to you go to a doctor (family physician or specialist)?**

| ⃝ | More than once a month |
| --- | --- |
| ⃝ | About every 1-3 months |
| ⃝ | About every 6 months |
| ⃝ | About every year |
| ⃝ | Less than once a year |

- 1. **Which mean of communication do you use to contact your physician when you cannot go in person?**

| ▢ | Via phone call |
| --- | --- |
| ▢ | Via text message (e.g.: SMS, Whatsapp) |
| ▢ | Via video call (e.g.: Whatsapp, Skype) |
| ▢ | Through web portal |

- 1. **Do you use any monitoring devices for your condition?**

| ▢ | Glucose meter |
| --- | --- |
| ▢ | Blood pressure monitor |
| ▢ | Electrocardiogram (ECG) |
| ▢ | None of the above |
| ▢ | Other |

- 1. **Are the monitoring devices you employ easy to use?**

| ▢ | Yes |
| --- | --- |
| ▢ | No |

- 1. **Please state the problems and/or limits you encountered.**

*The survey is anonymous; do not insert your personal information (e.g. name, surname, contact information)*

- 1. **Which of the following measurements would you judge more useful for your daily life?**

| ▢ | Blood pressure |
| --- | --- |
| ▢ | Heart rate |
| ▢ | Glycaemia |
| ▢ | Electrocardiogram |
| ▢ | House temperature |
| ▢ | Air pollution |
| ▢ | Noise exposure |
| ▢ | Social interaction frequency |
| ▢ | Diet habits |
| ▢ | Fall detection |
| ▢ | Mood |
| ▢ | Sleep |
| ▢ | Motor activity |
| ▢ | None of the above |

## A3 – Relationship with technology

- 1. **Describe your experience with the following devices:**
     - Smartphone
     - Smart TV (TVs that allow you to benefit from online services and interactivity with other smart devices)
     - Smartwatch (smart watches that have additional features, like step counter, heart rate monitor, and interactivity with the smartphone)
     - Smart lamp (Ambient lighting that automatically adjusts based on your preferences and allows voice interaction)
     - Smart thermostat (thermostats that automatically adjust based on your preferences and allow voice interaction)

| ⃝ | ⃝ | ⃝ | ⃝ | ⃝ | ⃝ |
| --- | --- | --- | --- | --- | --- |
| None | Very negative | Negative | Indifferent | Positive | Very positive |

- 1. **How useful do you find the use of technology in your daily activities?**

| ⃝ | Obstructive |
| --- | --- |
| ⃝ | Indifferent |
| ⃝ | Useful |
| ⃝ | Very useful |
| ⃝ | Fundamental |

- 1. **How easy do you find it to use technology?**

| ⃝ | Very difficult |
| --- | --- |
| ⃝ | Difficult |
| ⃝ | Medium |
| ⃝ | Easy |
| ⃝ | Very easy |

- 1. **How inclined are you to adopt a new technology, if you find it useful?**

| ⃝ | None at all |
| --- | --- |
| ⃝ | Little |
| ⃝ | Indifferent |
| ⃝ | Sufficiently |
| ⃝ | Highly |

## A5 – About SMART BEAR

- 1. **The SMART BEAR platform could regularly send you reports about your health status. Is this something that you would be interested in?**

| ⃝ | Yes |
| --- | --- |
| ⃝ | No |

- 1. **How often would you like to receive these periodic reports?**

| ⃝ | Monthly |
| --- | --- |
| ⃝ | Weekly |

- 1. **How would you like to receive these periodic reports?**

| ▢ | Via text message |
| --- | --- |
| ▢ | Via voice message |
| ▢ | Email |

- 1. **Who else would you like to have access to the periodic reports about your health status?**

| ▢ | Referring physician |
| --- | --- |
| ▢ | Spouse/partner |
| ▢ | Son/daughter |
| ▢ | Caregiver |
| ▢ | No one |
| ▢ | Other |

- 1. **The SMART BEAR platform could provide you with regular notifications or suggestions based on its observations. For example, “Your blood pressure is regularly higher than the normal for the last 2 weeks. You should visit your referring physician”. Is this something you might be interested in?**

| ⃝ | Yes |
| --- | --- |
| ⃝ | No |

- 1. **How inclined are you to follow the suggestions provided by such a platform?**

| ⃝ | None at all |
| --- | --- |
| ⃝ | Little |
| ⃝ | Indifferent |
| ⃝ | Sufficiently |
| ⃝ | Highly |

- 1. **How would you prefer to receive these suggestions?**

| ▢ | Via text message |
| --- | --- |
| ▢ | Via voice message |
| ▢ | Email |

- 1. **Who else would you like to have access to the suggestions about your health status?**

| ▢ | Spouse/partner |
| --- | --- |
| ▢ | Son/daughter |
| ▢ | Caregiver |
| ▢ | No one |
| ▢ | Other |

- 1. **Would you participate to a project that implements state-of-the-art technologies in your daily life, like SMART BEAR?**

| ⃝ | Yes |
| --- | --- |
| ⃝ | No |

- 1. **What would your main expectations be about the use of a platform such as SMART BEAR?**

| ▢ | To gain more autonomy in my daily activities |
| --- | --- |
| ▢ | To increase the sense of safety |
| ▢ | To improve the self-management of my health status |
| ▢ | To reduce the visits to my referring physician |
| ▢ | To improve my social life |
| ▢ | To save time |
| ▢ | To save money |
| ▢ | To improve my diet habits |
| ▢ | To gain self-esteem and self-confidence |
| ▢ | None of the above |
| ▢ | Other |

- 1. **What would your main concerns be about the use of a platform such as SMART BEAR?**

| ▢ | Data privacy |
| --- | --- |
| ▢ | Routine changes |
| ▢ | Erroneous information |
| ▢ | Erroneous notifications/suggestions |
| ▢ | Technical issues of the devices/Internet connection |
| ▢ | Education on Devices and Platform Usage |
| ▢ | Increased stress |
| ▢ | None of the above |
| ▢ | Other |

- 1. **Do you have any further comments? If you do, please add your comment in the box below.**

*The survey is anonymous; do not insert your personal information (e.g. name, surname, contacts)*

**If you are interested in participating to the SMART BEAR project, email to** [**smartbear@eservices4life.org**](mailto:smartbear@eservices4life.org)


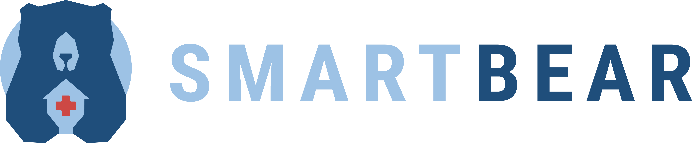


FG Older Adults

**Objectives of SMART BEAR**

The **SMART BEAR** research project, lead by 27 European partners (among which universities, research institutions and enterprises), is dedicated to people over 65 with at least two of the following medical conditions:

- - - Cardiovascular diseases
    - Imbalance
    - Hearing loss
    - Frailty
    - Cognitive decline
    - Mental health disorders

**SMART BEAR** intends to provide its users a technological support for the management of their own health in order to promote a healthy, independent life. SMART BEAR is composed of a technological platform, which will include smart devices like medical devices (e.g. smart blood pressure monitor), wearables (e.g. smartwatch to monitor physical activity) and smart home systems (e.g. smart lamps that can be controlled by voice or simply by clapping hands) to guarantee the continuous monitoring during the users’ daily lives. Through a dedicated App, the recorded data will be employed to provide personalized suggestions to keep in good health, to support the physicians in their decision making and ameliorate the patients’ therapeutic progress.

**Objectives of the questionnaire**

We are interested in your opinion about the functionalities proposed by the **SMART BEAR**

platform in order to understand the potential users’ preferences.

A few stories will be shown to help understand what the platform might offer. We kindly ask you to answer to the questions in complete honesty.

Thank you,

*The researchers from Fondazione Centro San Raffaele*

1

#
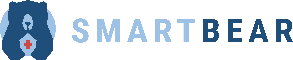
The stories


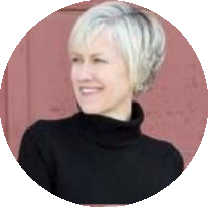

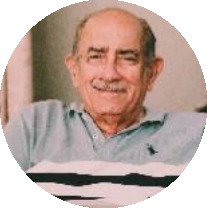


| **Carlo**  **Age**: 74  **Occupation**: Retired **Lives with**: Daughter **Hobby**: Playing cards |
| --- |
| **Description**  Carlo is a friendly and optimistic person by nature, over-indulgent in the pleasures and little to concerns. His daughter is alarmed by his continuous gain in weight and blames his **sedentary lifestyle** and the few extra treats that he allows himself in his **diet**. Moreover, at every measurement **the blood pressure is too high**. The physician urges him to intervene on his lifestyle, starting from a healthy diet and a better control and awareness over his health status. |
| **Health concerns**   - Sedentary lifestyle - Unhealthy diet habits - High blood pressure |
| **He wishes…**   - To keep in good health - To be independent |
| **He does not wish…**   - To weigh over his daughter |

| **Lidia**  **Age**: 65  **Occupation**: Housekeeper **Lives with**: Husband **Hobby**: Going to the theatre |
| --- |
| **Description**  Lidia is a sunny and day-dreaming person. Lately, she does not recognize herself in these qualities because she is very worried for her husband’s poor health, of whom she takes care of daily. Because of the related stress, **she feels very anxious and depressed**. By night, she has a hard time sleeping, and during the day **she is having difficulties with her memory and her attention**. She begins neglecting herself and her house, exposing herself and her husband to an **unhealthy environment**. Her mood and the gradual **hearing loss**, that she is experiencing lately, lead to loose her friends. Determined to overcome the adversities, she convinces herself to look for help and turning to a specialist. |
| **Health concerns**   - Low mood - Memory and attention difficulties - Hearing loss - Unhealthy environment |
| **She wishes…**   - To ameliorate mood and sleep - To build trust with her physician - Healthy environment |
| **She does not wish…**   - To isolate herself socially - To be stigmatized for hearing loss |

2

#
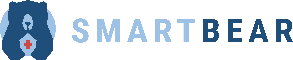
The physician’s plan


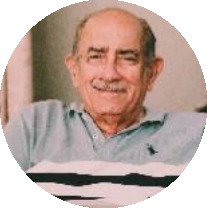

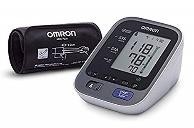

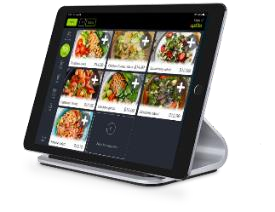

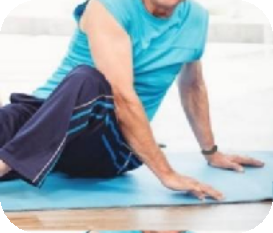


**Carlo**

- Sedentary lifestyle
- Unhealthy diet
- High blood pressure

**The physician’s plan for Carlo**

**Physical exercise program**

**Diet plan**

**Blood pressure**

**monitor**


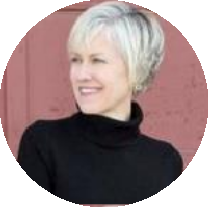

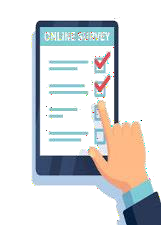

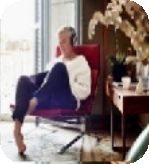

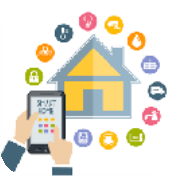

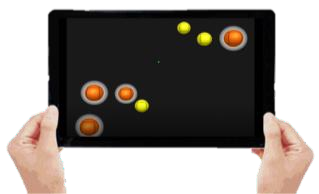

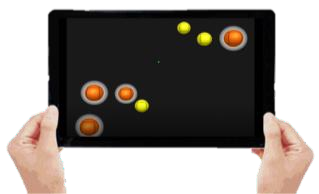

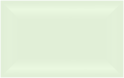

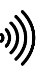

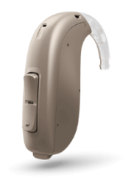


**Lidia**

- Low mood
- Memory and attention difficulties
- Hearing loss
- Unhealthy environment

**The physician’s plan for Lidia**

**Psychoeducational Mood monitoring**

**intervention**

**Cognitive training**

**Hearing training**

**Environment monitor**

3


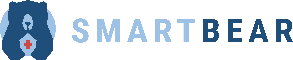


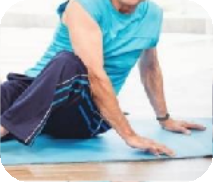

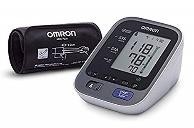

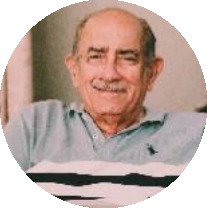


# SMART BEAR interventions for Carlo

**Carlo**

- Sedentary lifestyle
- Unhealthy diet
- High blood pressure

The physician’s plan for Carlo:

- **To follow a physical activity plan;**
- **To follow a diet plan;**
- **To monitor the blood pressure.**

**PHYSICAL ACTIVITY PLAN:** From the App, Carlo consults the plan and follows **guided exercises by video**. Moreover, the App **records the physical activity** he **carried on and provides real-time vocal instructions** to improve the exercise.

| (1) *Do you find it useful to have a guide to follow a physical activity plan?* | Yes | No |
| --- | --- | --- |
| (2) *Do you think this functionality is useful to that purpose?* | Yes | No |
| (3) *Would you want this functionality?* | Yes | No |
| (4) *Do you think you would be able to learn to use it?* | Yes | No |


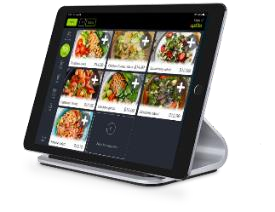
**DIET PLAN:** From the App, Carlo can **consult the clinician’s suggestions on the diet**. Moreover, he can record what he has eaten by simply taking a picture of his meal or by selecting it from a list.

| (1) *Do you find it useful to keep track of the diet?* | Yes | No |
| --- | --- | --- |
| (2) *Do you think this functionality is useful to that purpose?* | Yes | No |
| (3) *Would you want this functionality?* | Yes | No |
| (4) *Do you think you would be able to learn to use it?* | Yes | No |

**PHYSIOLOGICAL PARAMETERS MONITORING:** Carlo uses a «smart» blood pressure, that

**automatically records all the measurements** on the platform

| (1) *Do you find it useful to keep track of the physiological*  *parameters?* | Yes | No |
| --- | --- | --- |
| (2) *Do you think this functionality is useful to that purpose?* | Yes | No |
| (3) *Would you want this functionality?* | Yes | No |
| (4) *Do you think you would be able to learn to use it?* | Yes | No |

4


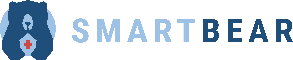


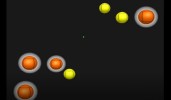

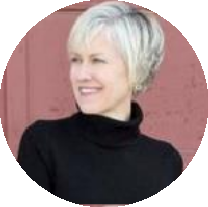


# SMART BEAR interventions for Lidia

**Lidia**

- Low mood
- Memory and attention difficulties
- Hearing loss
- Unhealthy environment

The clinician’s plan for Lidia:

- **To monitor her mood and provide a psycho-educational intervention;**
- **To conduct cognitive training;**
- **To conduct hearing training;**
- **To monitor the environment.**


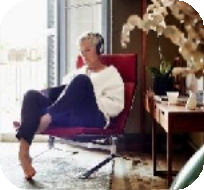
**PSYCHOEDUCATIONAL INTERVENTION:** Lidia accesses the App and **selects the topic that she would like to read or listen**. Those contents provide information and suggestions **to reach psychological wellbeing.**

| (1) *Do you find it useful to educate yourself about your*  *psychological concerns?* | Yes | No |
| --- | --- | --- |
| (2) *Do you think this functionality is useful to that purpose?* | Yes | No |
| (3) *Would you want this functionality?* | Yes | No |
| (4) *Do you think you would be able to learn to use it?* | Yes | No |


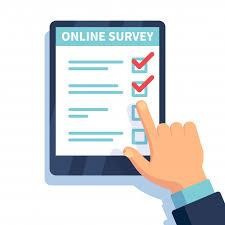
**MOOD MONITORING:** Lidia **fills in the questionnaires through the App** assigned by her psychologist to **monitor her mood, her leisure activities and her participation to the social life**.

| (1) *Do you find it useful to keep track of your mental wellbeing?* | Yes | No |
| --- | --- | --- |
| (2) *Do you think this functionality is useful to that purpose?* | Yes | No |
| (3) *Would you want this functionality?* | Yes | No |
| (4) *Do you think you would be able to learn to use it?* | Yes | No |


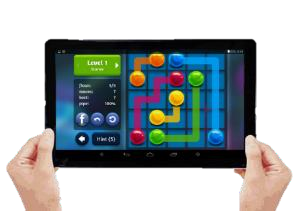
**COGNITIVE TRAINING:** Lidia uses the App to **exercise her memory and her attention through games by interacting with the touch-screen**. The results of each session are recorded and memorized by the App.

| (1) *Do you find it useful to be able to monitor and exercise your memory and attention?* | Yes | No |
| --- | --- | --- |
| (2) *Do you think this functionality is useful to that purpose?* | Yes | No |
| (3) *Would you want this functionality?* | Yes | No |
| (4) *Do you think you would be able to learn to use it?* | Yes | No |

5


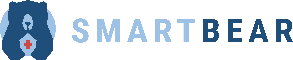


# SMART BEAR interventions for Lidia

**Lidia**

- Low mood
- Memory and attention difficulties
- Hearing loss
- Unhealthy environment

The clinician’s plan for Lidia:

- **To monitor her mood and provide a psycho-educational intervention;**
- **To conduct cognitive training;**
- **To conduct hearing training;**
- **To monitor the environment.**


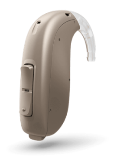
**HEARING TRAINING:** Lidia conducts the **guided exercises to train her hearing and listening comprehension through the App**.


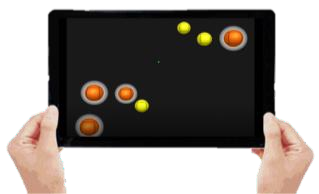

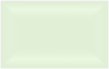

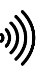

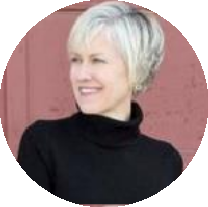


| (1) *Do you find it useful to be able to regularly exercise your hearing and listening comprehension?* | Yes | No |
| --- | --- | --- |
| (2) *Do you think this functionality is useful to that purpose?* | Yes | No |
| (3) *Would you want this functionality?* | Yes | No |
| (4) *Do you think you would be able to learn to use it?* | Yes | No |


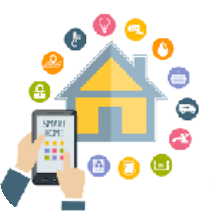
**ENVIRONMENT MONITORING:** The smart home devices that were given to Lidia are able to **monitor the indoor air quality, the temperature, the lighting and the noise exposure**. The vocal assistant can warn Lidia that the ambient is poorly lit or that the noise is too excessive for her hearing, or **automatically adjust some environmental conditions** through windows, thermostats and smart lamps.

| (1) *Do you find it useful to have a guide to live in a healthier environment?* | Yes | No |
| --- | --- | --- |
| (2) *Do you think this functionality is useful to that purpose?* | Yes | No |
| (3) *Would you want this functionality?* | Yes | No |
| (4) *Do you think you would be able to learn to use it?* | Yes | No |

6


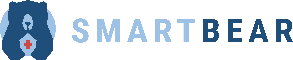


# The SMART BEAR functionalities


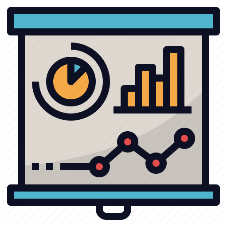

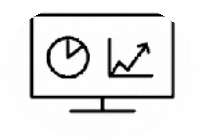


Carlo and Lidia can access and **visualize the data recorded by SMART BEAR at any time**. This includes the indexes of their health status, graphs that show their performance in the exercises and the tests through time and indexes of the progress in the programs.

***Visualization of recorded data***

| (1) *Do you find it useful to be able to consult the data recoded and analyzed by the platform?* | Yes | No |
| --- | --- | --- |
| (2) *Would you want this functionality?* | Yes | No |

| (1) *Do you find it useful to receive incentives to complete the*  *program?* | Yes | No |
| --- | --- | --- |
| (2) *Would you want this functionality?* | Yes | No |

7


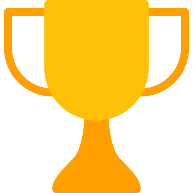

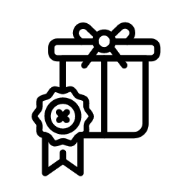


Carlo and Lidia follow a plan designed by their clinician for their training (physical,

cognitive or hearing). Every time they complete a session of exercises, they win a

«virtual reward» as an incentive for a better adherence to the program. They can always check **their progress in the planned exercises**.

***Incentives for a better adherence to the program***


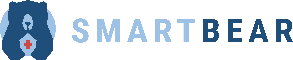


# The SMART BEAR functionalities


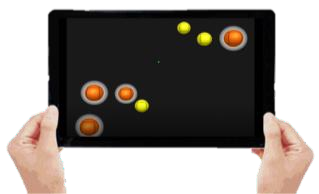

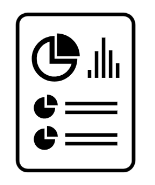


SMART BEAR can **send periodic reports** (monthly or weekly, via written or vocal messages) which summarize what has been recorded in that period regarding the health measurements and their activities. These reports **can be automatically sent directly to the user’s clinician**.

***Periodic reports***

| (1) *Do you find it useful to receive periodic health reports?* | Yes | No |
| --- | --- | --- |
| (2) *Would you want this functionality?* | Yes | No |

| (1) *Do you find it useful that your doctor also receives the periodic health reports?* | Yes | No |
| --- | --- | --- |
| (2) *Would you want this functionality?* | Yes | No |

| (1) *Do you find it useful to receive personalized suggestions regarding your health status?* | Yes | No |
| --- | --- | --- |
| (2) *Would you want this functionality?* | Yes | No |

| (1) *Do you find it useful to receive personalized reminders for the measurements and activities to perform?* | Yes | No |
| --- | --- | --- |
| (2) *Would you want this functionality?* | Yes | No |

8


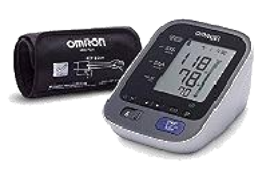

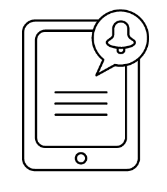


SMART BEAR can provide **personalized reminders and suggestions to improve one’s health status management**, in case it registers trends in the measurements that deserve the doctor’s attention.

***Personalized reminders and suggestions***


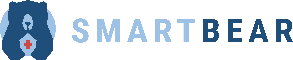


# The SMART BEAR functionalities

***Access to the health data for the caregivers***

If Carlo and Lidia give consent, their closest relative or **their caregiver can access the SMART BEAR App through their smartphone**. This consent enables one or more of these functions: access to all the recorded data, reception of the personalized suggestions and of the periodic reports.

| (1) *Do you find it useful that your relative has access to the data recorded by the platform?* | Yes | No |
| --- | --- | --- |
| (2) *Would you want this functionality?* | Yes | No |

| (1) *Do you find it useful that your relative receives the personalized*  *suggestions for you?* | Yes | No |
| --- | --- | --- |
| (2) *Would you want this functionality?* | Yes | No |

| (1) *Do you find it useful that your relative receives the periodic health reports?* | Yes | No |
| --- | --- | --- |
| (2) *Would you want this functionality?* | Yes | No |

| (1) *Do you find it useful to be able to book an appointment and consult your clinician remotely?* | Yes | No |
| --- | --- | --- |
| (2) *Would you want this functionality?* | Yes | No |

9


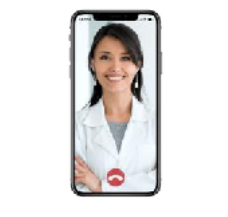

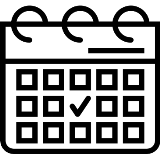

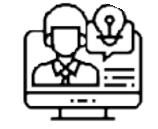


Carlo and Lidia **can book an appointment or a videocall with the specialist** by selecting a time from the availability calendar through the App.

***Remote consultation with the clinician***


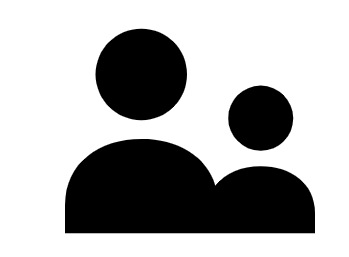

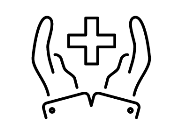


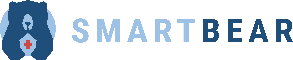


# SMART BEAR

The **SMART BEAR platform** provides the technological tools to support health self- management: through a personalized plan from the clinician, personalized suggestions and reminders, and all the functionalities shown here.


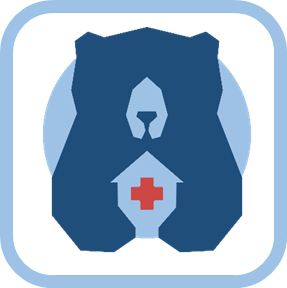


| (1) *Do you think the platform would be able to improve your awareness on your health status?* | Yes | No |
| --- | --- | --- |
| (2) *Do you think the platform could be able to help you manage your health problems?* | Yes | No |
| *(3) Do you think the platform could provide an objective support for the clinician’s decision making?* | Yes | No |
| *(4) Do you think the platform would be able to promote an active living,*  *physically and cognitively?* | Yes | No |
| *(5) Do you think the platform would be able to incentivize a healthy lifestyle?* | Yes | No |
| *(6) Do you think the platform could provide help to live a healthy, independent life?* | Yes | No |

10
