# Supplementary material for: Older Adults’ and Clinicians’ Perspectives on a Smart Health Platform for the Aging Population: Design and Evaluation Study
Source: JMIR Aging. 2022 Feb 28;5(1):e29623. doi: 10.2196/29623 (PMC8922154; doi:10.2196/29623)
Supplement: Multimedia Appendix 2 [file aging_v5i1e29623_app2.docx]

| *Experience with devices* | How do you evaluate your experience with the following devices?  (n=24) | |
| --- | --- | --- |
| *Ease of use* | How easy do you find technology to use?  (n=24) | \|  \| \|  \|  \|  \|  \|  \| \|  \|  \|  \|  \|  \|  \|  \|  \| \|  \|  \|  \|  \| \|  \|  \|  \|  \| \| \| --- \| --- \| --- \| --- \| --- \| --- \| --- \| --- \| --- \| --- \| --- \| --- \| --- \| --- \| --- \| --- \| --- \| --- \| --- \| --- \| --- \| --- \| --- \| --- \| --- \| --- \| --- \| \|  \| 4% \| \| \| \| \| \| 4% \| \| \| \| 54% \| \| \| \| \| 34% \| \| \| \| \| 4% \| \| \| \| \|  \| \| \|  \| Very difficult \| \| \| \| \| \| Difficult \| \| \| \| On average \| \| \| \| \| Easy \| \| \| \| \| Very easy \| \| \| \| \|  \| \| |
| *Usefulness* | How useful do you find technology for your daily activities? (n=24) | \|  \|  \| \|  \| \|  \| \|  \| \|  \| \|  \|  \|  \|  \|  \|  \|  \|  \|  \|  \|  \|  \|  \| \| --- \| --- \| --- \| --- \| --- \| --- \| --- \| --- \| --- \| --- \| --- \| --- \| --- \| --- \| --- \| --- \| --- \| --- \| --- \| --- \| --- \| --- \| --- \| --- \| \| 0% \| \| 8% \| \| 42% \| \| 29% \| \| 21% \| \| \| Obstructive \| \| Indifferent \| \| Useful \| \| Very useful \| \| Fundamental \| \| |
| *Willingness to adopt a new technology* | How inclined are you to use a new technology if considered useful?  (n=24) | \|  \| \|  \|  \|  \|  \| \|  \|  \|  \|  \|  \| \|  \|  \|  \|  \| \|  \|  \|  \|  \|  \|  \|  \|  \| \| --- \| --- \| --- \| --- \| --- \| --- \| --- \| --- \| --- \| --- \| --- \| --- \| --- \| --- \| --- \| --- \| --- \| --- \| --- \| --- \| --- \| --- \| --- \| --- \| --- \| --- \| \|  \| 0% \| \| \| \| \| 8% \| \| \| \| \| \| 0% \| \| \| \| \| 54% \| \| \| \| \| 38% \| \| \| \| \|  \| \|  \| Not at all \| \| \| \| \| Little \| \| \| \| \| \| Indifferent \| \| \| \| \| Sufficiently \| \| \| \| \| Highly \| \| \| \| \|  \| |

**Multimedia Appendix 2**
